# Breed- and Parity-Associated Incidence and Manifestation of Metabolic Disorders in Holstein and Jersey Cows During the Postpartal Transition Period

**DOI:** 10.3390/ani16060887

**Published:** 2026-03-12

**Authors:** Gi-Won Park, Seungmin Ha, Tai-Young Hur, Seogjin Kang, Chan-Lan Kim, Ui-Hyung Kim, Sang-Ik Oh, Mooyoung Jung

**Affiliations:** 1 Dairy Science Division, National Institute of Animal Science, Rural Development Administration, Cheonan 31000, Republic of Korea; rldnjs5731@gmail.com (G.-W.P.); tyohur@korea.kr (T.-Y.H.); vetchan@korea.kr (C.-L.K.); 2 College of Veterinary Medicine, Jeonbuk National University, Iksan 54596, Republic of Korea; sioh@jbnu.ac.kr; 3 Doctor Ha Animal Clinic, Gurye 57641, Republic of Korea; hyungyuha@hanmail.net; 4 Division of Animal Diseases & Health, National Institute of Animal Science, Rural Development Administration, Wanju 55365, Republic of Korea; hijin@korea.kr; 5 Poultry Research Institute, National Institute of Animal Science, Rural Development Administration, Pyeongchang 25342, Republic of Korea; uhkim@korea.kr

**Keywords:** Holstein, Jersey, ketosis, hypophosphatemia, hypocalcemia, hypomagnesemia

## Abstract

Dairy cows commonly experience negative energy balance during the periparturient period, predisposing them to metabolic disorders such as ketosis (KET), hypophosphatemia (HP), hypocalcemia (HC), and hypomagnesemia (HM). However, comparative data on breed- and parity-related differences remain limited. Therefore, these differences were evaluated in this study during the postpartal transition period. A total of 174 cows (149 Holstein, 25 Jersey) were monitored, and blood samples were collected from calving to 21 days postpartum at 3-day intervals to measure *β*-hydroxybutyrate, inorganic phosphorus, calcium, and magnesium concentrations. Metabolic disorders were defined using established thresholds. Data were analyzed using Fisher’s exact test, the Mann–Whitney U test, and generalized estimating equations. HP was the most prevalent disorder in both breeds. Jerseys had 2.83 times higher odds of KET, whereas Holsteins had 4.98 times higher odds and an earlier onset of HM. Multiparous Holsteins showed higher incidences of HP, HC, and HM compared to primiparous ones, while parity effects were minimal in Jerseys. Breed and parity significantly influenced both the incidence and onset timing of postpartal metabolic disorders. These findings highlight the importance of breed- and parity-specific health management strategies in dairy cattle farms.

## Simple Summary

A total of 149 Holstein and 25 Jersey cows were monitored, and blood samples were collected from calving to 21 days after parturition at 3-day intervals. *β*-hydroxybutyrate, inorganic phosphorus, calcium, and magnesium concentrations were measured to evaluate the metabolic disorders during the postpartal period. The incidence of metabolic disorders and the concentrations of metabolites were compared by breed and parity. Jerseys were more prone to ketosis, whereas Holsteins were more susceptible to hypomagnesemia and showed stronger parity-related trends. These findings highlight the importance of breed- and parity-specific health management strategies in dairy cattle farms.

## 1. Introduction

Ketosis (KET) occurs when cows present insufficient gluconeogenic precursors relative to the rate of fatty acid catabolism during negative energy balance [1,2], resulting in elevated blood ketone bodies, such as acetoacetate and *β*-hydroxybutyrate (BHBA) [3]. This disorder impairs appetite, milk production, and reproductive performance [4]. During late gestation, phosphorus requirements increase substantially, from 1.9 g/day at 190 days to 5.4 g/day at 280 days, to support fetal development [5]. This demand predisposes cows to hypophosphatemia (HP), characterized by decreased blood inorganic phosphorus (IP) [5,6]. Phosphorus homeostasis is closely related to calcium homeostasis because 1, 25-(OH)_2_D_3_ stimulates the intestinal absorption of both minerals [7]. HP can cause alert downer syndrome and is often complicated by concurrent hypocalcemia (HC) and hypomagnesemia (HM), potentially leading to postparturient hemoglobinuria [6,8]. HC results from decreased serum calcium (CA) concentration driven by increased milk production during early lactation [9,10]. HC is associated with dystocia, uterine prolapse, retained placenta, endometritis, infertility, and KET because of decreased smooth muscle function [11,12]. HM, defined as decreased serum magnesium (MG) concentrations, is caused by insufficient dietary MG supply and impaired absorption [9]. HM is associated with HC, decreased feed intake and milk yield, and may present with neurological symptoms (i.e., ataxia and tetanic muscle spasm) [13,14].

Although the incidence and risk factors of these metabolic disorders have been widely reported, information on the effects of breed and parity remains limited. Some previous studies have suggested that Jersey cows have a higher incidence of KET and HC than Holstein cows [15,16], and multiparous cows are more susceptible to HC than primiparous cows [17]. However, comparative data remains scarce or inconsistent. Jersey cattle are gaining attention in South Korea because of their thermal tolerance, disease resistance, concentrated milk yield, and adaptability [18], suggesting that physiological differences between breeds could influence the incidence and manifestation of metabolic disorders in the circumstance of South Korea. Furthermore, the effect of parity on these disorders may differ between breeds.

This study tested two hypotheses: (1) Jersey cows exhibit incidences and manifestations of postpartal metabolic disorders similar to Holstein cows; and (2) within each breed, primiparous and multiparous cows show similar incidences and manifestations of these disorders. The aim was to identify metabolic disorders, excluding the secondary cases from other disorders, during the postpartal transition periods of Holstein and Jersey cows raised on the same farm and to evaluate their incidence and characteristics by breed and parity.

## 2. Materials and Methods

### 2.1. Animals

This study included Holstein and Jersey cows raised and calved at the farm of the National Institute of Animal Science in Cheonan, Republic of Korea, from January 2018 to December 2024 (ethical approval number: NIAS-2018039). The farm houses approximately 360 cows and calves and produces about three tons of milk daily from around 90 lactating cows under free-stall barns. The farm facilities were well-ventilated and kept non- or mild-heat stressed (72 or less on the temperature–humidity index) throughout the testing period. Cows were fed a total mixed ration ad libitum, consisting of concentrates, soybean meal, corn silage, alfalfa hay, timothy hay, enzymes, minerals, and vitamin supplements (Appendix A). Throughout the study period, the management (e.g., diet, housing, bedding) practices remained consistent.

Prepartum, all dams received 5 mL of vitamin AD_3_E (Vigantol E, Elanco Animal Health, Inc., Greenfield, IN, USA) intramuscularly twice (Vitamin A 1.5 × 10^6^ IU, Vitamin D_3_ 5 × 10^5^ IU, and Vitamin E 250 mg were contained in one injection), at 6 and 3 weeks before the expected calving date. Cows with premature deliveries, abortions, stillbirths, or twin births were excluded. In addition, cows that were milked fewer than two times per day or that had mastitis, agalactia, theileriosis, acidosis, abomasal ulcers, or that were culled or medicated during the study period were excluded. In total, 174 cows (149 Holsteins and 25 Jerseys) met the inclusion criteria.

### 2.2. Blood Sampling and Analysis

Blood samples were collected once from the jugular vein of the cows at 6–23 h postpartum on the calving date, and then every 3 days until 21 days postpartum (eight time points in total). Sampling was performed in the morning, immediately after milking and prior to feeding. Blood samples were collected in serum-separating tubes.

BHBA concentrations were measured using handheld electronic meters (FreeStyle Optimum Neo, Abbott Diabetes Care Ltd., Witney, UK), which were calibrated or purchased annually, and *β*-ketone test strips (FreeStyle Optimum H *β*-ketone, Abbott Diabetes Care Ltd., Witney, UK) as described previously [19]. Samples were immediately transported to an on-site laboratory. Serum was separated by centrifuging at 1500× *g* for 10 min and stored at –70 °C until analysis. All biochemical analyses were performed on a single day using an automated biochemistry analyzer (Hitachi 7180, Hitachi Ltd., Tokyo, Japan). IP, CA, and MG concentrations were measured using commercial enzymatic assay kits (Fujifilm Wako Pure Chemical Ltd., Osaka, Japan) following calibration and quality control procedures.

### 2.3. Case and Group Definitions

Metabolic disorders were defined using the highest BHBA concentration and the lowest IP, CA, and MG concentrations recorded within 21 days of calving. The thresholds were as follows: normoketotic (highest BHBA < 1.2 mmol/L) and KET (highest BHBA ≥ 1.2 mmol/L); normophosphatemic (lowest IP ≥ 4.0 mg/dL) and HP (lowest IP < 4.0 mg/dL); normocalcemic (lowest CA ≥ 8.0 mg/dL) and HC (lowest CA < 8.0 mg/dL); and normomagnesemic (lowest MG ≥ 1.8 mg/dL) and HM (lowest MG < 1.8 mg/dL) [20,21]. Cows were categorized as primiparous and multiparous according to parity at parturition.

### 2.4. Statistical Analysis

Statistical analyses were performed using the Statistical Package for the Social Sciences (SPSS version 27.0; IBM Corp., Armonk, NY, USA). Fisher’s exact test was used to analyze cross-tabulations of breed or parity with the presence of metabolic disorders at the cow level (a cow was classified once as a case if the threshold was met at any postpartal time point). The odds ratios for each disorder were also calculated by this Fisher’s exact test. A Mann–Whitney U test was used to compare age, parity, and day of onset of metabolic disorders between breeds or parity groups because of unequal group sizes. An exchangeable generalized estimating equation model was only used to evaluate repeated measurements of BHBA, IP, CA, and MG concentrations between breeds or parity groups, with time and group specified as fixed effects. A sequential Bonferroni’s post hoc test for the generalized estimating equation model was used to compare the concentrations of BHBA, IP, CA, and MG between the cow breeds and parities at each time point. Data are presented as mean ± standard deviation, and statistical significance was set at *p* < 0.05 for all analyses.

## 3. Results

### 3.1. Comparison of Metabolic Disorders by Breed

**Figure 1:**
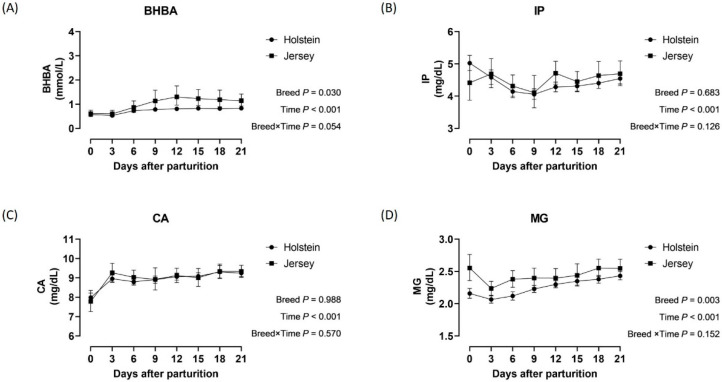
Concentrations of *β*-hydroxybutyrate (**A**), inorganic phosphorus (**B**), calcium (**C**), and magnesium (**D**) during the postpartal transition period according to each breed. Abbreviations: BHBA, *β*-hydroxybutyrate; IP, inorganic phosphorus; CA, calcium; MG, magnesium. Notes: Black circles and squares show the average concentrations in Holstein and Jersey cows, respectively. Upper and lower horizontal bars to the mean points represent the 95% confidence intervals. *p*-values shown at the bottom right of each panel were obtained from the generalized estimating equation model.

The average age and parity of Holstein cows were 4.27 ± 1.97 years and 2.44 ± 1.28, respectively, and those of Jersey cows were 4.20 ± 1.66 years and 2.16 ± 1.14, indicating comparable age and parity distributions between breeds. The incidence rate and days of onset for each metabolic disorder by breed are summarized in Table 1. The additional information regarding the heat stress at parturition and the clinical cases are supplied in Appendix B. HP was the most prevalent disorder in both breeds. The odds of KET were 2.83-fold higher in Jerseys than in Holsteins (*p* = 0.030), whereas the odds of HM were 4.98-fold higher in Holsteins than in Jerseys (*p* = 0.026). In addition, HM onset occurred significantly earlier in Holsteins than in Jerseys (*p* = 0.007). Mean changes in BHBA, IP, CA, and MG concentrations during the postpartal period are shown in Figure 1. The average BHBA and MG concentrations were 0.41 mmol/L and 0.18 mg/dl higher, respectively, in Jerseys than in Holsteins throughout the 21-day period. No significant breed-by-time interaction was observed for BHBA, IP, CA, or MG concentrations.

### 3.2. Comparison of Metabolic Disorders by Parity in Holstein and Jersey Cattle

The incidence rate and day of onset of metabolic disorders by parity in Holstein cows are presented in Table 2. Multiparous Holsteins had a higher incidence of all metabolic disorders except KET, compared to primiparous Holsteins. There were no significant differences in the day of onset for any metabolic disorder between the two parity groups in Holstein cows. In Jersey cows, the incidence rate and day of onset of metabolic disorders by parity are shown in Table 3. Overall, incidence and onset were similar between primiparous and multiparous Jerseys. However, multiparous Jerseys showed a marginally later onset of KET than primiparous Jerseys (*p* = 0.050).

**Figure 2:**
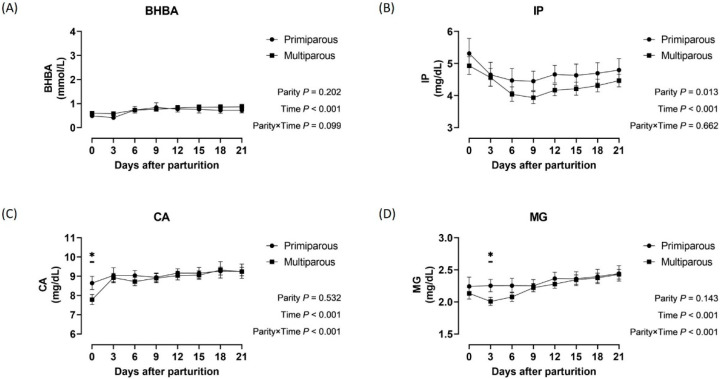
Concentrations of *β*-hydroxybutyrate (**A**), inorganic phosphorus (**B**), calcium (**C**), and magnesium (**D**) during the postpartal transition period by parity of Holstein cows. Abbreviations: BHBA, *β*-hydroxybutyrate; IP, inorganic phosphorus; CA, calcium; MG, magnesium. Notes: Black circles and squares show the average concentrations in primiparous and multiparous Holstein cows, respectively. Upper and lower horizontal bars to the mean points represent the 95% confidence intervals. *p*-values shown at the bottom right of each panel were obtained from the generalized estimating equation model. The significant differences at each time point in post hoc analysis (*p* < 0.05) are indicated by asterisks (*).

**Figure 3:**
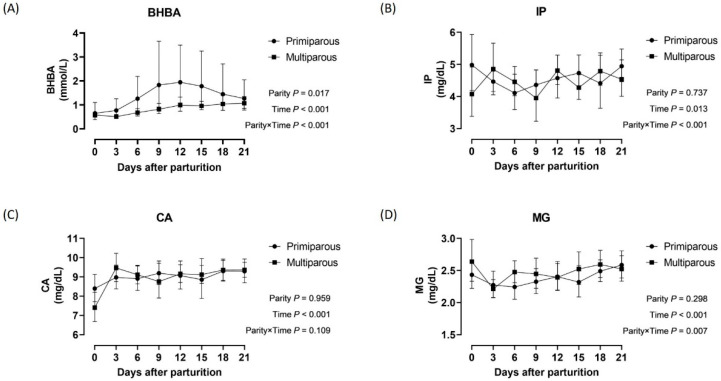
Concentrations of *β*-hydroxybutyrate (**A**), inorganic phosphorus (**B**), calcium (**C**), and magnesium (**D**) during the postpartal transition period by parity of Jersey cows. Abbreviations: BHBA, *β*-hydroxybutyrate; IP, inorganic phosphorus; CA, calcium; MG, magnesium. Notes: Black circles and squares show the average concentrations in primiparous and multiparous Jersey cows, respectively. Upper and lower horizontal bars to the mean points represent the 95% confidence intervals. *p*-values shown at the bottom right of each panel were obtained from the generalized estimating equation model.

Time-serial changes in BHBA, IP, CA, and MG concentrations during the postpartal period in primiparous and multiparous Holsteins are shown in Figure 2. The average IP concentrations in primiparous Holsteins were 0.34 mg/dL higher than those in multiparous Holsteins. A significant parity-by-time interaction was observed for CA (*p* < 0.001) and MG concentrations (*p* < 0.001) in Holsteins. The multiparous Holsteins presented significantly lower concentrations of CA at parturition (*p* < 0.05) and MG at 3 days after parturition, respectively. Figure 3 presents the postpartal changes in BHBA, IP, CA, and MG concentrations in primiparous and multiparous Jersey cows. Primiparous Jerseys had average BHBA concentrations 0.93 mmol/L higher than multiparous Jerseys. Significant parity-by-time interactions were observed for BHBA (*p* < 0.001), IP (*p* < 0.001), and MG concentrations (*p* = 0.007) in Jerseys.

## 4. Discussion

This study investigated the incidence and timing of four major metabolic disorders—KET, HP, HC, and HM—during the postpartal transition period in Holstein and Jersey cows and assessed their relationships with breed and parity. We expected that these findings would provide insight into breed- and parity-specific vulnerabilities that may contribute to the development of metabolic disorders in early lactation.

Jersey cows had a higher incidence of KET than Holsteins did, which coincided with higher BHBA concentrations during the postpartal period. Numerous previous studies have reported a higher incidence of KET in Jerseys, but direct comparisons of blood BHBA concentrations between breeds have been limited [15,22,23]. KET reflects an inadequate adaptive response to negative energy balance that occurs when the hepatic capacity to oxidize or export non-esterified fatty acids is exceeded [20]. BHBA also plays a regulatory role in milk fat synthesis, and Jerseys are typically known to produce milk with higher fat content than Holsteins do [24,25]. For this reason, these inherent differences in fatty acid catabolism may contribute to their increased KET susceptibility. In addition, primiparous Jerseys exhibited earlier onset and higher BHBA concentrations than multiparous Jerseys did in this study, which was consistent with the previous finding that young Jerseys may be particularly prone to KET [26]. However, this interpretation should be considered as a possible explanation because the mechanistic biomarkers (e.g., glucose, non-esterified fatty acids, liver enzymes) were not measured in this study.

The results of this study presented that most cows experience HP within the first few weeks postpartum, consistent with a previous study [27]. However, there is still limited information about breed-dependent differences in HP incidence to the best of our knowledge. In this study, Holstein and Jersey cows showed similar HP incidence and onset; however, the associated risk factors could differ between breeds [5]. In Holsteins, parity at parturition was associated with HP incidence and IP concentrations, and multiparous cows had higher incidences of both HP and HC. In Jerseys, parity did not significantly influence HP or HC incidence. Because phosphorus homeostasis is closely linked to calcium homeostasis, these findings suggest that the interaction between HP and HC may be more pronounced in Holsteins than in Jerseys.

The overall incidence of HC in this study was similar to that reported in a previous study [21]. Contrary to earlier reports that suggested a higher HC incidence in Jerseys [16,28], no significant difference in HC incidence between Holsteins and Jerseys was detected in this study. This discrepancy may be because of differences in diagnosis method as many previous studies focused on the symptoms, whereas the present study evaluated the serum Ca concentrations. In a previous report, the probability of adapting different thresholds for subclinical HC according to the cow breed has been suggested [29], which could affect the result of this study. In Holsteins, HC incidence increased with age and parity, and many multiparous cows already had CA concentrations below the HC threshold on the day of calving, which is consistent with earlier evidence that clinical and subclinical HC increase with parity [17,30,31]. However, in Jerseys, parity did not significantly affect HC incidence, consistent with some previous findings [32].

The overall incidence of HM in this study was slightly higher than in a previous study [33], which may be explained by repeated sampling rather than a single time point. Although a prior study reported similar HM incidence and serum MG concentrations between Holsteins and Jerseys [34], our findings showed higher incidence of HM in Holsteins than in Jerseys. This difference may reflect breed-related variation in ruminal magnesium absorption, which is critical for maintaining magnesium homeostasis [35]. Holsteins have been reported to have a slightly higher ruminal pH than Jerseys, which may decrease magnesium solubility and absorption and thereby increase the risk of HM [36]. However, this should be interpreted only as a potential explanation because ruminal pH was not estimated in this study. In Holsteins, the factors of increasing age and parity were associated with decreased MG concentrations and higher HM incidence, consistent with previous reports that magnesium absorption declines with age [9,37]. However, the insufficient number of HM cases in Jerseys precluded the evaluation of parity effects in this breed. Therefore, further studies are needed to clarify HM risk factors across breeds.

A key limitation of this study is the relatively small number of Jersey cows, reflecting the limited distribution of this breed in South Korea and the single-farm setting. This limited Jersey sample size may have decreased statistical power from some breed- and parity-level comparisons. Also, the parity-stratified odds ratios should be interpreted cautiously because of the sparse number of cows, particularly for Jersey cows. Nevertheless, the uniform management conditions decreased confounding and allowed for the clearer assessment of breed differences.

## 5. Conclusions

To the best of our knowledge, this is the first field study to simultaneously compare the incidence of four major metabolic disorders and their associations with age and parity in Holstein and Jersey cows during the postpartal transition period. Jerseys had 2.83 times higher odds of KET, whereas Holsteins had 4.98 times higher odds and earlier onset of HM. Multiparous Holsteins showed higher incidences of HP, HC, and HM compared to primiparous ones, while parity effects were minimal in Jerseys. While the pronounced parity effects observed in Holsteins provide robust insights, the corresponding findings in Jerseys should be considered preliminary due to the limited sample size. These results could assist veterinarians and producers, particularly in regions where Jerseys are newly introduced, in developing breed- and parity-specific strategies to prevent and manage postpartal metabolic disorders, while highlighting the need for larger-scale studies to confirm the Jerseys’ risk factors.

## Figures and Tables

**Table 1:** Comparison of incidence and days of onset postpartum between Holstein and Jersey cows.

		KET	HP	HC	HM
Number of cows	Holstein (149 cows)	71 (47.7)	117 (78.5)	68 (45.6)	45 (30.2)
	Jersey (25 cows)	18 (72.0)	18 (72.0)	15 (60.0)	2 (8.0)
	OR (Jersey/Holstein)	2.82 (1.11–7.16)	0.70 (0.27–1.83)	1.79 (0.75–4.23)	0.20 (0.05–0.89)
	*p*-value	0.030 *	0.448	0.201	0.026 *
Days of onset postpartum	Holstein	10.06 ± 6.21	5.54 ± 6.01	1.59 ± 3.38	2.33 ± 3.25
	Jersey	9.50 ± 5.07	5.67 ± 6.33	0.20 ± 0.78	13.30 ± 2.12
	*p*-value	0.967	0.680	0.088	0.007

Abbreviations: KET, ketosis; HP, hypophosphatemia; HC, hypocalcemia; HM, hypomagnesemia; OR, Odds ratio; * *p* < 0.05. Notes: The data for number of cows are expressed as number of cows (percentages of the same breed cows), OR (95% confidence interval), and *p*-values which were calculated by Fisher’s exact test. The data in days of onset postpartum are expressed as the mean ± standard deviations. The *p*-values in days of onset postpartum are calculated by the Mann–Whitney U test.

**Table 2:** Comparison of incidence and days of onset postpartum between primiparous and multiparous Holstein cows.

		KET	HP	HC	HM
Number of cows	Primiparous Holsteins (37 cows)	15 (40.5)	23 (62.2)	9 (24.3)	3 (8.1)
	Multiparous Holsteins (112 cows)	56 (50.0)	94 (83.9)	59 (52.7)	42 (37.5)
	OR (Multiparous/Primiparous)	1.47 (0.69–3.12)	3.18 (1.38–7.32)	3.46 (1.50–8.00)	6.80 (1.97–23.52)
	*p*-value	0.348	0.010 *	0.004 *	<0.001 *
Days of onset postpartum	Primiparous Holstein	11.40 ± 5.58	4.96 ± 4.11	3.33 ± 6.08	6.00 ± 10.39
	Multiparous Holstein	9.70 ± 6.37	5.68 ± 5.21	1.32 ± 2.75	2.07 ± 2.25
	*p*-value	0.388	0.802	0.420	1.000

Abbreviations: KET, ketosis; HP, hypophosphatemia; HC, hypocalcemia; HM, hypomagnesemia; OR, Odds ratio; * *p* < 0.05. Notes: The data for number of cows are expressed as number of cows (percentages of the same breed cows), OR (95% confidence interval), and *p*-values which were calculated by the Fisher’s exact test. The data in days of onset postpartum are expressed as the mean ± standard deviations. The *p*-values in days of onset postpartum are calculated by the Mann-Whitney U test.

**Table 3:** Comparison of incidence and days of onset postpartum between primiparous and multiparous Jersey cows.

		KET	HP	HC	HM
Number of cows	Primiparous Jerseys (10 cows)	9 (90.0)	7 (70.0)	5 (50.0)	1 (10.0)
	Multiparous Jerseys (15 cows)	9 (60.0)	11 (73.3)	10 (66.7)	1 (6.7)
	OR (Multiparous/Primiparous)	0.17 (0.02–1.68)	1.18 (0.20–6.93)	2.00 (0.39–10.31)	0.64 (0.04–11.63)
	*p*-value	0.179	1.000	0.442	1.000
Days of onset postpartum	Primiparous Jersey	7.00 ± 5.20	6.86 ± 5.67	0.00 ± 0.00	12 ^a^
	Multiparous Jersey	12.00 ± 3.67	4.91 ± 6.88	0.30 ± 0.95	15 ^a^
	*p*-value	0.050	0.211	0.768	NA ^a^

Abbreviations: KET, ketosis; HP, hypophosphatemia; HC, hypocalcemia; HM, hypomagnesemia; OR, Odds ratio; NA, not available. ^a^ Statistical analysis was not conducted for HM onset because only one HM case was observed in each parity group. Notes: The data for number of cows are expressed as number of cows (percentages of the same breed cows), OR (95% confidence interval), and *p*-values which were calculated by Fisher’s exact test. The data in days of onset postpartum are expressed as the mean ± standard deviations. The *p*-values in days of onset postpartum are calculated by the Mann–Whitney U test.

## Data Availability

The raw data supporting the conclusions of this article will be made available by the authors on request.

## Abbreviations

The following abbreviations are used in this manuscript:

KET	Ketosis
BHBA	*β*-hydroxybutyrate
HP	Hypophosphatemia
IP	Inorganic Phosphorus
HC	Hypocalcemia
HM	Hypomagnesemia
CA	Calcium
MG	Magnesium

## Appendix A

The composition of diet, which were maintained consistently according to NRC/NASEM recommendations for transition cows.

**Table A1:** Ingredient composition of the total mixed ration fed to the cows.

Items	Ingredient Composition (kg per Cow)
Concentrate	5.5
Alfalfa hay	2.0
Timothy	2.0
Tall fescue	6.0
Beet pulp	1.5
Soybean meal	0.5
Whole cottonseed	2.0
Corn silage	12.0
Vitamin premix	0.5
Trace minerals	0.5

**Table A2:** Chemical composition of the total mixed ration fed to the cows.

Items	Chemical Composition (% of Dry Matter)	
	Total Mixed Ration Without Concentrate	Concentrate
Dry matter	95.8	89.7
Crude protein	15.0	22.1
Soluble crude protein	4.3	7.9
Neutral detergent insoluble crude protein	3.9	2.3
Acid detergent insoluble crude protein	1.3	0.8
Crude fiber	26.2	7.7
Amylase-treated neutral detergent fiber	54.2	30.9
Acid detergent fiber	32.8	11.9
Lignin	5.6	3.2
Starch	8.6	22.7
Ether extract	3.4	4.4
Ash	9.4	7.1
Calcium	0.8	1.0
Phosphorus	0.5	0.6
Net energy for milking (MJ/kg)	5.9	7.2

## Appendix B

All details regarding the heat stress at parturition (seasonal based) and the detailed classification about the metabolic disorders are expressed in this section.

**Table A3:** The detailed classification about the seasonal based heat stress (HS) at parturition in Holstein and Jersey cows.

		KET	HP	HC	HM
Holstein	None HS & None disorder	66 (44.3)	22 (14.8)	65 (43.6)	77 (51.7)
	None HS & Metabolic disorder	50 (33.6)	94 (63.1)	51 (34.2)	39 (26.2)
	HS & None disorder	12 (8.1)	10 (6.7)	16 (10.7)	27 (18.1)
	HS & Metabolic disorder	21 (14.1)	23 (15.4)	17 (11.4)	6 (4.0)
Jersey	None HS & None disorder	7 (28.0)	6 (24.0)	8 (32.0)	18 (72.0)
	None HS & Metabolic disorder	13 (52.0)	14 (56.0)	12 (48.0)	2 (8.0)
	HS & None disorder	0 (0.0)	1 (4.0)	2 (8.0)	5 (20.0)
	HS & Metabolic disorder	5 (20.0)	4 (16.0)	3 (12.0)	0 (0.0)

Notes: The cows which delivered dams in June, July and August were classified into heat stress classification and the others were classified into non-HS classification. Data are expressed as numbers (percentages of the same breed cows). Abbreviations: HS, heat stress; KET, ketosis; HP, hypophosphatemia; HC, hypocalcemia; HM, hypomagnesemia.

**Table A4:** The detailed classification about the clinical cases in Holstein and Jersey cows.

		KET	HP	HC	HM
Holstein	None disorder	78 (52.3)	32 (21.5)	81 (54.4)	104 (69.8)
	Subclinical disorder	51 (34.2)	111 (74.5)	60 (40.3)	43 (28.9)
	Clinical disorder	20 (13.4)	6 (4.0)	8 (5.4)	2 (1.3)
Jersey	None disorder	7 (28.0)	7 (28.0)	10 (40.0)	23 (92.0)
	Subclinical disorder	10 (40.0)	17 (68.0)	14 (56.0)	2 (8.0)
	Clinical disorder	8 (32.0)	1 (4.0)	1 (4.0)	0 (0.0)

Notes: Normoketotic (highest BHBA < 1.2 mmol/L), subclinical KET (highest BHBA ≥ 1.2 and < 3.0 mmol/L), and clinical KET (highest BHBA ≥ 3.0 mmol/L); normophosphatemic (lowest IP ≥ 4.0 mg/dL), subclinical HP (lowest IP < 4.0 and ≥ 2.0 mg/dL), and clinical HP (lowest IP < 2.0 mg/dL); normocalcemic (lowest CA ≥ 8.0 mg/dL), subclinical HC (lowest CA < 8.0 and ≥ 5.6 mg/dL), and clinical HC (lowest CA < 5.6 mg/dL); normomagnesemic (lowest MG ≥ 1.8 mg/dL), subclinical HM (lowest MG < 1.8 and ≥ 1.1 mg/dL), and clinical HM (lowest MG < 1.1 mg/dL). Abbreviations: KET, ketosis; HP, hypophosphatemia; HC, hypocalcemia; HM, hypomagnesemia; BHBA, β-hydroxybutyrate; IP, inorganic phosphorus; CA, calcium; MG, magnesium.
